# Characteristics of international primary care practices and physicians related to advance care planning: a cross-sectional survey study

**DOI:** 10.1186/s12875-023-02103-8

**Published:** 2023-07-14

**Authors:** Molly A. Nowels, David Nowels, Julia Sheffler, Hillary D. Lum

**Affiliations:** 1 Department of Health Behavior, Society, and Policy, Rutgers School of Public Health, Piscataway, NJ USA; 2 Institute for Health, Health Policy, and Aging Research, Rutgers University, New Brunswick, NJ USA; 3 Department of Family Medicine, University of Colorado School of Medicine, Aurora, CO USA; 4 Center for Translational Behavioral Science, Department of Behavioral Sciences and Social Medicine, Florida State University College of Medicine, Tallahassee, FL USA; 5 Division of Geriatric Medicine, Department of Medicine, University of Colorado School of Medicine, Aurora, CO USA

**Keywords:** Advance care planning, Primary care providers, Palliative care

## Abstract

**Background:**

Primary care providers (PCPs) are well-situated to delivery primary palliative care such as advance care planning (ACP). The aim of this work is to identify practice characteristics, including features found in advanced primary care models (APCMs), that predict PCP engagement of patients in ACP.

**Methods:**

We analyzed characteristics of physician respondents and their practices associated with ACP conversations in older and sicker patients using data from 11 countries who participated in the 2015 Commonwealth Fund International Survey of Primary Care Physicians in 10 Nations. The primary outcome was how routinely these ACP conversations are reported. We used a validated measure to describe practice-level characteristics of advanced primary care models. We conducted bivariate and multivariable analyses to determine PCP and practice characteristics associated with routinely engaging patients in ACP and with documenting patient preferences in medical records.

**Results:**

Respondents (N = 12,049) predominantly were older than 45 and did not view their jobs as high stress. PCPs reported routinely engaging patients in ACP work in practices with more APCM features. They are more likely to view their jobs as high stress, to work more hours, to practice in rural areas, and to work in smaller practices. Multivariable analyses showed that older PCP age, higher perceived stress of the job, practice location in rural areas, and increased number of APCM features were associated with more ACP conversations. Increased number of APCM features was also associated with higher odds of routinely recording patient preferences in their medical records.

**Conclusions:**

In this international survey, physician and practice characteristics, including having features associated with APCMs, were associated with whether physicians routinely discuss ACP with patients who are older and sicker. Many features of APCMs may facilitate ACP discussions between PCPs and patients.

**Supplementary Information:**

The online version contains supplementary material available at 10.1186/s12875-023-02103-8.

## Background

Advance care planning (ACP) is a proactive communication process supporting adults at any age or health status to understand and share their personal values, life goals, and preferences for future medical care. ACP helps to better align patient preferences and treatments for people with serious and chronic illness [1]. Patients near the end of life and their families have better health care experiences if they have engaged in ACP. Specifically, patients report greater concordance between their wishes and the health care they receive [2]. Additionally, family members report lower stress and depression symptoms [2, 3]. The COVID-19 pandemic has highlighted the importance of ACP [4, 5].

Primary care providers (PCPs) are well-suited to have ACP discussions [6–9] due to the longitudinal, patient centered, and comprehensive relationships they have with patients. These discussions improve patient satisfaction about their care [10]. The ability to engage patients in ACP discussions is an important skill for PCPs, however, these discussions are not a systematic or routine part of practice, even with older and sicker patients [8]. Between 61% and 91% of frail older adults would like to have ACP discussions, but only 2–29% have had such conversations [11]. Thus, it is important to understand the characteristics that increase the likelihood of ACP conversations with PCPs.

Evolving advanced primary care models (APCMs) incorporate features such as patient-team partnership, comprehensiveness and care coordination, continuity of care, and team-based care [12]. These APCMs help to ensure that patients receive appropriate and timely treatment, features particularly relevant for engaging patients in ACP. In the US, the most notable example of an APCM is the Patient Centered Medical Home (PCMH). PCMH practices demonstrate higher quality and value of care provided, particularly among patients with complex medical needs [13, 14]. PCPs practicing in a PCMH report that they provide palliative care more than their non-PCMH counterparts [15]. Similar models are in different stages of development internationally [16].

We have previously described an association between how often United States (US) PCPs reported engaging their patients in ACP conversations with aspects of their practice, including the number of PCMH-like features found in their practices [9]. This work was limited to a US sample. It remains unknown whether practice characteristics of APCM in different international contexts similarly support PCP engagement of patients in ACP. In the current study, we investigate practice characteristics, inclusive of, but not limited to, those of APCM, associated with ACP in an international context. We hypothesized that practice qualities of APCMs found in an international sample were associated with routine engagement of patients in ACP discussions.

## Methods

### Data source

Data from the 2015 Commonwealth Fund International Health Policy Survey of Primary Care Physicians in 10 Nations was used to perform analyses. This survey consists of physician responses from nationally representative random samples of 12,049 PCPs in 11 countries: Australia, Canada, Germany, France, Netherlands, New Zealand, Norway, Sweden, Switzerland, United States, and United Kingdom. The Commonwealth Fund survey is an International Health Policy survey collecting nationally representative data in Organization for Economic Cooperation and Development (OECD) countries to compare features of health system performance. The sample included general practitioners, family physicians, internists, and pediatricians. Responses were collected online, by mail, or by phone. Complete survey data and methods are available [17]. This study was approved by the Colorado Multiple Institutional Review Board.

### Outcome variable

Physicians were asked “Do you have conversations with older or sicker patients about the health care treatment they want or do not want in the event they become very ill or injured, and cannot make decisions for themselves?” The primary ACP outcome, an indicator for ACP conversations, was created by grouping responses as “yes, routinely” versus other responses. In secondary analyses we created a binary outcome to reflect whether PCPs who routinely engage patients in ACP also routinely document their patients’ preferences resulting from these conversations.

### Independent variables

Independent variables included physician characteristics and primary care practice characteristics. Physician variables included the following: age (younger than 45 years vs. 45 years or greater), sex, and whether the physician considered their work to be high stress (“Extremely” and “Very” vs. “Somewhat”, “Not too”, or “Not at all” stressful).

Practice characteristics included location (rural, small town, suburb, urban), and how many full-time equivalent (FTE) physicians were employed at the practice, with categories based on quartiles of the distribution of responses (1-1.45 FTE, 1.5–2.95 FTE, 3-5.95 FTE, and 6-100 FTE). One survey question asked how many hours per week physicians worked, and we created a four-category variable for hours worked, with cut points at each quartile of the distribution of hours worked (0–34 h/week, 35–40 h/week, 40–49 h/week, 50–80 h/week). Finally, we also created a four-category variable using a question from the survey regarding how long the PCP’s typical visits lasted, with cut points based on quartiles of the distribution. Length of visit categories included 1–11 min, 12–14 min, 15–19 min, and 20–240 min.

### Assessment of APCM

To assess the impact of PCMH-like qualities that influence ACP in this sample, we used a validated, 41-item PCMH index which we previously developed using the sample of US physician respondents from this same international dataset [9]. According to the Bodenheimer model there are 10 building blocks to high-performing, advanced, primary care practices: engaged leadership, data-driven improvement, empanelment, team-based care, patient-team partnership, population management, continuity of care, prompt access to care, comprehensiveness and care coordination, and a template of the future to allow for fewer and longer in-person visits [12]. Briefly, to create the index we used descriptive items reported about the practice from the survey. From these practice descriptors items to be considered for the PCMH-index were selected through a 2-round Delphi process. Panelists for the Delphi process were primary care researchers familiar with logic models of PCMH practices. After 2 rounds of the Delphi process items were removed if they did not meet an item-total cutoff of 0.20 for inclusion in the index. The index had a Cronbach’s alpha value of 0.879. This index was then validated against a survey question asked of US respondents to the survey: whether the physician practiced in a PCMH. Because the data used to create this index were secondary data, this index does not include items for every single building block of the Bodenheimer model. Our team and Delphi panelists felt that this index has question items specific to 5 of the 10 building blocks with components of questions reflecting 8 of the building blocks.

An unadjusted logistic regression was performed with the index as the predictor and binary PCMH question as the outcome. The results from this model found an odds ratio of 1.11 (95% CI: [1.09, 1.13], p < 0.001), indicating that every additional item in the index that a respondent endorsed was associated with an 11% increase in the odds of the practice being a PCMH. Each item in the PCMH index represents a practice feature characteristic of practices that have PCMH designation. Further details for the derivation of this index can be found in prior work [9]. Higher index scores (ranging from 0 to 41) mean that the practice has more features characteristic of and associated with a US PCMH. The average index score in US practices from which the index was derived was 23.7 (SD 7.7).

### Data analysis

Bivariate analyses were performed using chi-square tests. For the PCMH scale, a continuous variable, we performed a two-sample t-test. Missing values were excluded from these analyses. Next, single random imputation was used for items missing less than 10% of responses. These were randomly identified based on the range of responses to each item. We then performed a series of mixed-effects logistic regression models using the PCMH index to predict the ACP outcome. We allowed for a random intercept for country to account for within-country clustering. First, we used an unadjusted model including only the PCMH index to predict ACP. In the survey, respondents from two countries (Sweden and Switzerland) were not asked for their age or sex. Therefore, two adjusted models were performed. First, an adjusted model with all covariates except for physician age and physician sex was performed, including respondents from Sweden and Switzerland. Next, a model with all covariates, including physician age and sex was used, excluding respondents from Sweden and Switzerland. Adjusted models included the imputed independent variables. Because the relationship between ACP and APCMs has been established in the US sample from these data, [9] we performed sensitivity analyses including all countries except the US.

Finally, among the subgroup of PCPs who endorsed routine ACP, we performed an analysis focusing on whether patient preferences are recorded in their medical records. We used both unadjusted and adjusted logistic regressions with random a random intercept for country to investigate the effect of the PCMH scale on documentation of preferences.

All models were performed using R version 5.3.1 and the *glmer* function from the *lme4* R package [18]. Two-sided p-values less than 0.05 were considered statistically significant. Age group and gender had high rates of missing responses because Swedish and Swiss respondents were not asked these questions.

## Results

### Descriptive analyses

The majority of respondents, (N = 7,116 (59.8%)) do not endorse routine ACP conversations with patients, while 4,793 (40.2%) do (Table 1). The mean PCMH index score is 24.0 (SD 6.3). The mean index score is higher for PCPs routinely engaging in ACP with patients vs. those who did not. In bivariate analyses (Table 1), PCPs who report routinely engaging patients in ACP are more likely to view their jobs as high stress, to be in the highest quartile of hours worked, to practice in rural areas, to be the lower two quartiles of number of physicians in the practice, and to be in the lowest quartile for time per visit.

**Table 1:** Bivariate Associations of International Physicians and Practice Characteristics with ACP.

Variable		Characteristic	Total	Routine ACP, N (%)	No Routine ACP, N (%)	p-value
		Total	12,049	(N = 4793, 40.2%)	(N = 7116, 59.8%)	
	Physician-Reported Characteristics					
Age Group		Younger than 45	2815 (34.9%)	1155 (32.0%)	1643 (37.5%)	< 0.001
		45 or older	5246 (65.1%)	2455 (68.0%)	2737 (62.5%)	
Gender		Female	3452 (42.9%)	1436 (39.9%)	1984 (45.3%)	< 0.001
		Male	4595 (57.1%)	2163 (60.1%)	2393 (54.7%)	
Stress		Not high stress	7310 (61.0%)	2827 (59.4%)	4404 (62.2%)	0.002
		High stress	4667 (39.0%)	1935 (40.6%)	2679 (37.8%)	
	Practice-Level Characteristics					
Hours Worked		0–34 h/week	2920 (24.6%)	946 (20.1%)	1933 (27.6%)	< 0.001
		35–40 h/week	3239 (27.3%)	1127 (23.9%)	2069 (29.5%)	
		40–49 h/week	1826 (15.4%)	662 (14.1%)	1150 (16.4%)	
		50–80 h/week	3871 (32.7%)	1974 (41.9%)	1861 (26.5%)	
Practice Location		City	4577 (38.3%)	1687 (35.4%)	2827 (40.0%)	< 0.001
		Suburb	2321 (19.4%)	927 (19.5%)	1360 (19.2%)	
		Small Town	2993 (25.0%)	1199 (25.2%)	1770 (25.1%)	
		Rural	2072 (17.3%)	951 (20.0%)	1108 (15.7%)	
Number of Physicians in Practice		1-1.45	2580 (22.2%)	1133 (24.2%)	1408 (20.7%)	< 0.001
		1.5–2.95 FTE	2236 (19.3%)	983 (21.0%)	1225 (18.0%)	
		3-5.95 FTE	3776 (32.6%)	1427 (30.5%)	2317 (34.1%)	
		6-100 FTE	3008 (25.9%)	1134 (24.2%)	1845 (27.2%)	
Time per Clinic Visit		1-11 min	2461 (20.6%)	1318 (27.8%)	1125 (16.0%)	< 0.001
		12–14 min	720 (6.04%)	333 (7.02%)	381 (5.40%)	
		15–19 min	3497 (29.3%)	1401 (29.5%)	2058 (29.2%)	
		20–240 min	5251 (44.0%)	1693 (35.7%)	3486 (49.4%)	
PCMH Index		Mean (SD)	24.0 (6.30)	25.3 (6.15)	23.2 (6.25)	< 0.001

### Multivariable analyses

In an unadjusted model (Table 2), the APCM index has an odds ratio of 1.055, indicating that every additional feature of a APCM in a PCP’s practice is associated with an effect size of 5.5% higher odds of the PCP routinely engaging patients in ACP discussions (OR: 1.055, 95% CI: [1.048, 1.062]). After adjustment for all covariates except for PCP gender and age, every additional feature of a APCM in a PCP’s practice is associated with 5.3% higher odds of the PCP routinely engaging patients in ACP discussions (OR: 1.053, 95% CI: [1.046, 1.06]). After full adjustment, including PCP gender and age, the increase in odds that the PCP engages patients in ACP discussions is 5.1% for every additional feature of a APCM (OR: 1.051, 95% CI: [1.043, 1.06]). The average PCP’s practice in this study had 24 APCM features, with 3.3 times higher odds of routinely engaging patients in ACP relative to PCPs in practices with 0 APCM features.

**Table 2:** Unadjusted, partially adjusted, and fully adjusted models of APCM index predicting ACP.

		Model 1: Unadjusted			Model 2: Partially Adjusted			Model 3: Fully adjusted		
		N = 11,909			N = 11,155			N = 7,595*		
		OR	95% CI	p-value	OR	95% CI	p-value	OR	95% CI	p-value
PCMH	PCMH Index	1.055	(1.048, 1.062)	< 0.001	1.053	(1.046, 1.06)	< 0.001	1.051	(1.043, 1.06)	< 0.001
Age	Younger than 45	--	--	--	--	--	--	REF	REF	REF
	45 or older				--	--	--	1.212	(1.09, 1.347)	< 0.001
Gender	Female				--	--	--	REF	REF	REF
	Male				--	--	--	1.049	(0.946, 1.164)	0.3610
Stress of job	Somewhat, not too, or not at all stressful				REF	REF	REF	REF	REF	REF
	Extremely or very stressful				1.198	(1.098, 1.308)	< 0.001	1.244	(1.118, 1.384)	< 0.001
h Worked	0–34 h/week				REF	REF	REF	REF	REF	REF
	35–40 h/week				1.073	(0.955, 1.205)	0.235	1.005	(0.873, 1.158)	0.942
	40–49 h/week				1.104	(0.966, 1.262)	0.147	1.047	(0.884, 1.238)	0.597
	50–80 h/week				1.582	(1.411, 1.774)	< 0.001	1.434	(1.247, 1.65)	< 0.001
Practice location	City				REF	REF	REF	REF	REF	REF
	Suburb				1.044	(0.93, 1.171)	0.468	1.122	(0.983, 1.282)	0.089
	Small town				1.215	(1.093, 1.351)	< 0.001	1.296	(1.139, 1.473)	< 0.001
	Rural area				1.538	(1.367, 1.731)	< 0.001	1.590	(1.375, 1.837)	< 0.001
Number of FTE doctors in practice	1-1.45				REF	REF	REF	REF	REF	REF
	1.5–2.95 FTE				1.047	(0.924, 1.186)	0.470	1.077	(0.926, 1.251)	0.337
	3-5.95 FTE				0.940	(0.832, 1.062)	0.320	0.942	(0.813, 1.09)	0.4218
	6-100 FTE				0.958	(0.841, 1.09)	0.514	1.017	(0.871, 1.188)	0.8304
Time per Clinic Visit	1-11 min				REF	REF	REF	REF	REF	REF
	12–14 min				0.910	(0.757, 1.095)	0.318	0.942	(0.778, 1.14)	0.538
	15–19 min				1.058	(0.919, 1.217)	0.434	1.104	(0.952, 1.281)	0.189
	20–240 min				1.142	(0.982, 1.328)	0.084	1.207	(1.022, 1.425)	0.027

*Fully adjusted model does not include respondents from Sweden and Switzerland, as they were not asked to report their age or sex

In the partially adjusted model, there are increased odds of routinely engaging patients in ACP discussion associated with the PCP finding their job very or extremely stressful (OR 1.198, 95% CI: [1.098, 1.308]), PCPs working the highest number of hours each week by quartile (OR: 1.582, 95% CI: [1.411, 1.774]), and PCPs practicing in small towns (OR:1.215, 95% CI: [1.093, 1.351]) or rural areas (OR:1.538, 95% CI: [1.367, 1.731]).

The fully adjusted model has the same significantly associated characteristics as the partially adjusted model, with similar effect sizes. However, the fully adjusted model also has significantly increased odds of engaging patients in ACP discussions for PCPs who are 45 years or older (OR:1.212, 95% CI: [1.09, 1.347]) and PCPs in the highest quartile of minutes spent per average visit with a patient (20–240 min) (OR:1.207, 95% CI: [1.022, 1.425]).

The sensitivity analysis, excluding the US sample, had similar findings. PCMH scale effect sizes of the models ranged from 1.042 (95% CI: 1.033, 1.051) in the fully adjusted model to 1.048 (95% CI: 1.042, 1.056) in the unadjusted model. Further sensitivity analysis results can be found in Table 1 in the Appendix.

### Subgroup analysis

In the subgroup analysis of PCPs who endorsed routinely having ACP conversations with their older and sicker patients, 89.9% endorsed that patient preferences are routinely recorded in their medical records. Average PCMH scale scores are 24.5 for those who do not routinely document preferences and 25.3 for those who do (two sample t-test p-value = 0.004). In both unadjusted and adjusted logistic regressions, each unit increase in the PCMH scale is associated with about a 2% increase in the odds of patient preferences being recorded in their medical records (all p < 0.05, see Appendix Table 2 for full results).

## Discussion

In this large representative sample of PCPs from 11 OECD countries, those working in practices with more features found in APCMs have higher odds of routinely engaging older or sicker patients in ACP discussions. While PCPs in practices with more characteristics reflecting APCMs have higher odds of engaging patients in ACP, other practice characteristics also impact whether PCPs engage patients in ACP. Specifically, those working in practices in a small town or rural area, those who work more hours per week, and those with longer appointments have higher odds of routine ACP with their patients. In addition, PCPs who are 45 years old or older and who view their jobs as very or extremely stressful also have a greater likelihood of routinely having ACP discussions with their patients. Finally, among PCPs who endorse routine ACP conversations, those in practices with more APCM features have higher odds of routinely documenting patient preferences in their medical records.

We believe these findings are broadly applicable to primary care practice internationally. The database used is composed of a large representative sample of PCPs from countries with differing social, economic, and health care delivery systems. Additionally, we used a APCM measure that was developed against a known advanced primary care practice model, the Patient Centered Medical Home. Our findings align with those previously reported among US physicians and practices [9].

Additionally, these findings are similar to research from Japan showing that features such as first contact, longitudinal relationships, coordination, comprehensiveness, and community orientation were associated with ACP discussions between clinicians and patients [19]. These attributes are common in models of APCMs. Other studies point to additional practice features common to APCMs including transferable electronic health records [20–22] team based care delivery, [8] and the ability to alter workflow to include routine ACP discussions [20].

Other practice features that were associated with ACP engagement may be aided through adoption of APCM features. For example, PCPs report that time pressure is a barrier to engaging patients in ACP discussions [20, 23–25]. Our analysis is consistent with those findings in that longer routine appointments are associated with higher odds that the PCP routinely engages patients in ACP discussions. We postulate that longer scheduled appointments may reduce the time pressure experienced by providers, facilitating ACP discussions. Alternatively, longer routine visits may be a proxy for treating older and sicker patients who require more time in a patient-PCP encounter. In this situation, the importance of ACP is even more relevant. Approaches such as introducing team-based care and using an adequate template for the future that includes modalities such as telehealth, visits with other team members, and group visits may alleviate some time pressure for PCPs and may improve documentation of ACP [26].

The current study also found a strong effect of location of practice on engaging patients in ACP discussions. This aligns with prior work [7, 15]. PCPs in small towns and rural communities may have to take on a wider range of responsibilities, including discussing ACP, than those working in cities where palliative care specialists are more abundant. Palliative care constitutes a small but important part of the workload of PCPs in rural areas [27, 28].

This study has some limitations. First, because we performed a secondary data analysis using an existing dataset, we are unable to account for unmeasured confounding. Next, not all questions were asked of respondents in all countries. Some of the covariates we selected were not well-populated in the database. Next, because primary care practice models and policies vary by country, these findings may not be generalizable outside of the countries included in the analysis. Finally, our study used an index of accepted components commonly seen in APCM delivery. However, we are limited in our assessment because not all attributes of APCMs are included in the dataset. To fully assess the impact of such advanced delivery models, standardized measures of all the attributes of high functioning primary care practices must be developed. If such data were available, models could be developed to determine which building blocks [12] are most important for PCPs to engage patients in ACP conversations.

To fully assess the impact of advanced delivery models on primary care engaging patients in ACP, measures of attributes of high functioning primary care practices beyond what we could evaluate using this database, should be developed and evaluated. We especially encourage evaluation of the impact of team-based approaches to ACP conversations in primary care practices. APCMs often include a variety of clinicians [12]. Social workers, behavioral health providers, and nurses all might skillfully engage patients and families in aspects of ACP with appropriate training and support. We found that PCPs with higher stress and longer visit times are more likely to routinely engage patients in ACP. Highly structured team-based care could help mitigate stress and distribute the time required to conduct such discussions among several clinicians. While some research is being conducted about the potential role of team based approach in primary care practices [29] additional evaluation will be important. Other attributes of APCM not included in the APCM index developed for this study (continuity, comprehensiveness) have been identified as being associated with patient reported ACP [30]. If such data were available, models could be developed to determine which building blocks [12] are most important for PCPs to engage patients in ACP conversations.

## Conclusion

Among OECD countries, PCPs working in practices with more qualities of an APCM have higher odds of routinely engaging their patients in ACP conversations. Each APCM quality a PCP’s practice is associated with a 5.1% increase in the odds of routinely engaging patients in ACP, confirming the study hypothesis. However, other practice features such as location and time spent per visit were also significantly associated with routinely engaging patients in ACP, indicating that there are other features of practice structure which also impact these conversations by physicians and may benefit from integrating certain APCM features. Among PCPs who routinely engage patients in ACP, those working in practices with more APCM qualities have higher odds of routinely documenting patient preferences for care.

## Electronic supplementary material

Below is the link to the electronic supplementary material.

**Supplementary Material 1**

**Supplementary Material 2**

**Supplementary Material 3**

## Data Availability

The data analyzed in this study are available by request from the Commonwealth Fund. Aggregate data and reporting on the data is available here: https://www.commonwealthfund.org/international-health-policy-center/system-profiles.

## Abbreviations

ACP: Advance care planning
APCM: Advanced primary care model
CI: Confidence Interval
OECD: Organization for Economic Cooperation and Development
OR: Odds ratio
PCMH: Patient centered medical home
PCP: Primary care provider
US: United States
