# Supplementary material for: Characteristics of international primary care practices and physicians related to advance care planning: a cross-sectional survey study
Source: BMC Prim Care. 2023 Jul 14;24:146. doi: 10.1186/s12875-023-02103-8 (PMC10347754; doi:10.1186/s12875-023-02103-8)
Supplement: Supplementary file 1 — Supplementary Material 1 [file 12875_2023_2103_MOESM1_ESM.docx]

**Appendix Table 1.** **Unadjusted, partially adjusted, and fully adjusted models of APCM index predicting ACP, excluding the US.**

|  |  | Model 1: Unadjusted | | | Model 2: Partially Adjusted | | | Model 3: Fully adjusted | | |
| --- | --- | --- | --- | --- | --- | --- | --- | --- | --- | --- |
|  |  | N=11,909 | | | N=11,155 | | | N=7,595* | | |
|  |  | OR | 95% CI | p-value | OR | 95% CI | p-value | OR | 95% CI | p-value |
| PCMH | PCMH Index | 1.049 | (1.042, 1.056) | <0.001 | 1.046 | (1.039, 1.054) | <0.001 | 1.042 | (1.033, 1.051) | <0.001 |
| Age | Younger than 45 | -- | -- | -- | -- | -- | -- | REF | REF | REF |
|  | 45 or older |  |  |  | -- | -- | -- | 1.218 | (1.089, 1.363) | 0.001 |
| Gender | Female |  |  |  | -- | -- | -- | REF | REF | REF |
|  | Male |  |  |  | -- | -- | -- | 0.99 | (0.886, 1.106) | 0.855 |
| Stress of job | Somewhat, not too, or not at all stressful |  |  |  | REF | REF | REF | REF | REF | REF |
|  | Extremely or very stressful |  |  |  | 1.188 | (1.084, 1.303) | <0.001 | 1.232 | (1.099, 1.382) | <0.001 |
| Hours Worked | 0-34 hours/week |  |  |  | REF | REF | REF | REF | REF | REF |
|  | 35-40 hours/week |  |  |  | 1.088 | (0.965, 1.227) | 0.166 | 1.029 | (0.887, 1.194) | 0.707 |
|  | 40-49 hours/week |  |  |  | 1.112 | (0.969, 1.277) | 0.131 | 1.068 | (0.895, 1.275) | 0.466 |
|  | 50-80 hours/week |  |  |  | 1.609 | (1.428, 1.813) | <0.001 | 1.491 | (1.285, 1.73) | <0.001 |
| Practice location | City |  |  |  | REF | REF | REF | REF | REF | REF |
|  | Suburb |  |  |  | 1.115 | (0.986, 1.261) | 0.083 | 1.246 | (1.077, 1.441) | 0.003 |
|  | Small town |  |  |  | 1.236 | (1.107, 1.379) | <0.001 | 1.337 | (1.167, 1.532) | <0.001 |
|  | Rural area |  |  |  | 1.595 | (1.411, 1.803) | <0.001 | 1.686 | (1.447, 1.963) | <0.001 |
| Number of FTE doctors in practice | 1-1.45 |  |  |  | REF | REF | REF | REF | REF | REF |
|  | 1.5-2.95 FTE |  |  |  | 1.078 | (0.946, 1.229) | 0.258 | 1.12 | (0.953, 1.317) | 0.168 |
|  | 3-5.95 FTE |  |  |  | 1.005 | (0.882, 1.144) | 0.946 | 1.029 | (0.877, 1.208) | 0.723 |
|  | 6-100 FTE |  |  |  | 1.030 | (0.896, 1.184) | 0.680 | 1.13 | (0.952, 1.341) | 0.162 |
| Time per Clinic Visit | 1-11min |  |  |  | REF | REF | REF | REF | REF | REF |
|  | 12-14 min |  |  |  | 0.893 | (0.739, 1.08) | 0.243 | 0.928 | (0.762, 1.13) | 0.456 |
|  | 15-19 min |  |  |  | 1.053 | (0.908, 1.22) | 0.494 | 1.105 | (0.945, 1.293) | 0.211 |
|  | 20-240 min |  |  |  | 1.069 | (0.911, 1.256) | 0.413 | 1.087 | (0.906, 1.303) | 0.369 |

*Fully adjusted model does not include respondents from Sweden and Switzerland, as they were not asked to report their age or sex.
