# Supplementary material for: Characteristics of international primary care practices and physicians related to advance care planning: a cross-sectional survey study
Source: BMC Prim Care. 2023 Jul 14;24:146. doi: 10.1186/s12875-023-02103-8 (PMC10347754; doi:10.1186/s12875-023-02103-8)
Supplement: Supplementary file 2 — Supplementary Material 2 [file 12875_2023_2103_MOESM2_ESM.docx]

Appendix Table 2. Effect of APCM on routine documentation of preferences in medical record following ACP.

|  |  | Model 1: Unadjusted | | | Model 2: Partially Adjusted | | |
| --- | --- | --- | --- | --- | --- | --- | --- |
|  |  | N=4,793 | | | N=4,531 | | |
| Variable | Term | OR | 95% CI | p-value | OR | 95% CI | p-value |
| PCMH | PCMH Index | 1.022 | (1.005, 1.038) | 0.009 | 1.020 | (1.003 ,1.037) | 0.024 |
| Stress | Somewhat, not too, or not at all stressful |  |  |  | REF | -- | -- |
|  | Extremely or very stressful |  |  |  | 1.009 | (0.809 ,1.259) | 0.937 |
| Hours | 0-34 hours/week |  |  |  | REF | -- | -- |
|  | 35-40 hours/week |  |  |  | 1.079 | (0.769 ,1.514) | 0.660 |
|  | 40-49 hours/week |  |  |  | 0.745 | (0.517 ,1.072) | 0.113 |
|  | 50-80 hours/week |  |  |  | 0.916 | (0.678 ,1.237) | 0.566 |
| Location | City |  |  |  | REF | -- | -- |
|  | Suburb |  |  |  | 0.995 | (0.744 ,1.332) | 0.973 |
|  | Small town |  |  |  | 0.994 | (0.751 ,1.315) | 0.967 |
|  | Rural area |  |  |  | 1.130 | (0.843 ,1.514) | 0.414 |
| Number of physicians | 1-1.45 FTE |  |  |  | REF | -- | -- |
|  | 1.5-2.95 FTE |  |  |  | 1.186 | (0.884 ,1.593) | 0.255 |
|  | 3-5.95 FTE |  |  |  | 1.088 | (0.806 ,1.467) | 0.582 |
|  | 6-100 FTE |  |  |  | 1.413 | (1.015 ,1.967) | 0.041 |
| Time per clinic visit | 1-11 min |  |  |  | REF | -- | -- |
|  | 12-14 min |  |  |  | 0.901 | (0.57 ,1.426) | 0.658 |
|  | 15-19 min |  |  |  | 1.029 | (0.733 ,1.445) | 0.869 |
|  | 20-240 min |  |  |  | 0.971 | (0.677 ,1.392) | 0.871 |
