# Supplementary material for: Characteristics of international primary care practices and physicians related to advance care planning: a cross-sectional survey study
Source: BMC Prim Care. 2023 Jul 14;24:146. doi: 10.1186/s12875-023-02103-8 (PMC10347754; doi:10.1186/s12875-023-02103-8)
Supplement: Supplementary file 3 — Supplementary Material 3 [file 12875_2023_2103_MOESM3_ESM.docx]

**Appendix List of questions in PCMH scale**

Question in 2015 Commonwealth Fund International Health Policy Survey of Primary Care Physicians in 10 Nations used to create the index of APCP/PCMH Scale

q3 - Your rating of changes in quality of medical care patients receive compared to 3 years previously?

q8 - Does your practice have an arrangement where patients can see a Dr or nurse if needed when the practice is closed, without going to hospital or ED?

Q9 - Do you/other personnel that work in the practice provide care in any of the following:

q9b - Coordinate f/u care with hospitals?

q9d - Coordinate care with social services or other community providers?

Q11. - How prepared is your practice to manage care for the following patients:

q11a - Chronic illness?

q11b - Mental health problems?

q11c - Substance use issues?

q11d - In need of palliative care?

q11e - In need of Long Term Care?

q11f - With community needs?

q11g - Needing language translation?

q11h - With dementia?

q12 - Does your practice use personnel to monitor and manage care for patients with chronic conditions that need regular follow up care?

q13 - Are pts with chronic conditions given written instructions about how to manage their own care at home?

q14 - For patients with chronic conditions, are their self-management goals recorded in their med record?

Q15 – When your patient has been seen by a specialist, how often do you receive the following?:

q15a - A report back with all relevant health info?

q15b - Info about changes specialist has made to med or care plan?

q15c - Info that is timely and available when needed?

Q16. - When your patient goes to the ED or is admitted to the hospital how often do you receive:

q16a - Notification seen in ED or admitted to hospital?

q16b - Notification being discharged from hospital?

q17 - After hospital dc how long does it take for you to get info needed to manage the patient?

!20. - If any of your patients receive home health services how often do:

q20a - You or practice personnel communicate with patient’s home care provider?

q20b - Are you advised of a relevant change in patient’s condition?

q21 - How easy or difficult is it to coordinate patient care with social services or other community providers? 2

Q29 - Can your practice generate information about your patients using computerized processes:

q29e - List of lab results for individual patient?

q29f - Clinical summary for each visit to give to the patient?

Q30.- Are the following routinely performed in your practice using computer:

q30a - Patient sent reminder notices about preventive or follow up care?

q30b - All lab tests followed until results reach clinicians?

q30c - You receive prompt to provide patient with test results?

q30d - You receive reminder about guideline-based intervention/ screening? 2

Q31. - Does the place where you practice routinely receive and review data about the following:

q31a - Clinical outcomes?

q31b. - Surveys of patient satisfaction?

q31c. - Patient hospital or emergency department use?

q31d.-Percent of patients receiving recommended care?

q32. Are any of your own clinical performance reviewed against targets at least annually?

q33. Do you receive info on how the clinical performance of your practice compares to other practices?
